# Regulatory signatures involved in the cell cycle pathway contribute to egg production heterosis in chicken

**DOI:** 10.1186/s40104-025-01156-2

**Published:** 2025-02-04

**Authors:** Jingwei Yuan, Yuanmei Wang, Yanyan Sun, Yunlei Li, Aixin Ni, Qin Li, Hanhan Yang, Xinying Xu, Yunhe Zong, Hui Ma, Jilan Chen

**Affiliations:** 1 Key Laboratory of Animal (Poultry) Genetics Breeding and Reproduction, Ministry of Agriculture and Rural Affairs, Institute of Animal Sciences, Chinese Academy of Agricultural Sciences, Beijing, 100193 China; 2 Shandong Provincial Key Laboratory of Animal Biotechnology and Disease Control and Prevention, College of Animal Science and Veterinary Medicine, Shandong Agricultural University, Taian, 271018 China

**Keywords:** Cell cycle, Chicken, Dominance, Egg production heterosis, Ovary transcriptome, WGCNA

## Abstract

**Background:**

Crossbreeding is widely promoted as an efficient strategy to improve the productivity in agriculture. The molecular mechanism underlying heterosis for egg production is always intriguing in chicken. The transcriptional dynamic changes play a crucial role in the formation of heterosis, but little is known for the egg production traits.

**Results:**

In present study, we measured the continuous manifestation of heterosis ranging from 2.67% to 10.24% for egg number in the crossbreds generated by reciprocal crossing White Leghorn and Beijing You chicken. The high-quality transcriptomes of ovary for purebreds (WW and YY) and crossbreds (WY and YW) in 5 laying stages were sequenced and integrated to identify regulatory networks relevant to the heterosis. We found highly conserved transcriptional features among 4 genetic groups. By using weighted gene co-expression network analysis (WGCNA), we obtained multiple gene co-expression modules that were significantly correlated with egg number for each group. The common KEGG pathways including apelin signaling pathway, cell cycle, ribosome, spliceosome and oxidative phosphorylation, were screened for the 2 crossbreds. Then, we identified consensus co-expression modules (CMs) that showed divergent expression pattern among crossbred (WY or YW) and purebreds (WW and YY). The hub genes of CMs were again overrepresented in the cell cycle pathway, and the crossbreds exhibited temporally complementary dominance of hub genes in the 5 laying stages. These results suggested that the crossbreds inherited from both parents to maintain the ovary function by cell cycle-related genes, contributing to the persistent heterosis for egg production. Furthermore, the dominant genes including *MAD2L1*, *CHEK2* and *E2F1* were demonstrated to function in ovarian follicle development and maturation and could be the candidate genes for egg production heterosis.

**Conclusion:**

Our study characterized the dynamic profile of genome-wide gene expression in ovary and highlighted the role of dominant expression of cell cycle pathway genes in heterosis. These findings provided new insights for the molecular mechanism of egg production heterosis, which would facilitate the rational choice of suitable parents for producing crossbred chickens with higher egg production.

**Supplementary Information:**

The online version contains supplementary material available at 10.1186/s40104-025-01156-2.

## Background

Heterosis is a phenomenon showing improvement in productivity, fertility and viability of hybrids over that of their parents [1]. It was widely exploited in commercial animal and plant breeding schemes in early days, even as far as back to 1893 in poultry [2]. The importance of heterosis utilization is evident in agriculture. If cultivating pure lines or breeds with superior characteristics or performance is a constant pursuit for breeders, the higher benefits from heterosis utilization make this pursuit more valuable and deserved when crossing breeds with complementary performance. The classic hypotheses for heterosis include the dominance hypothesis that assumes deleterious recessive alleles from one parental line was masked by dominant alleles in the other parental line, the overdominance as result of the advantageous combinations of alleles at heterozygous loci, and the epistasis effect from the interactions among loci result in the heterosis [3]. In addition, epigenetics [4], metabolite changes [5], function complementation among dominant loci [6] and single-parent expression [7] was demonstrated to contribute the heterosis in hybrid crops in recent decades.

Chicken is one of most successful poultry in utilizing heterosis, providing most of the high-quality animal protein for human beings. The hybrid vigor in egg and meat production has made studying the heterosis very allure and be always an ongoing hot topic in chickens. Using crosses between Cornish (meat-type chicken) and Rhode Island White (egg-type chicken), the formation of heterosis for growth traits and abdominal fat traits were attributed to the nonadditive expression of oxidative phosphorylation-related genes in the muscle [8] and to the over-dominant expressed gene *HMGCL* in the abdominal fat [9], respectively. The molecular mechanism of heterosis for sexual maturity [10] and feed efficiency traits [11] were also explored by analyzing the genome-wide gene expressions. Egg production is the most important component of selection indexes in laying chicken. Previously, Amuzu-Aweh et al. [12, 13] put forward squared difference in allele frequency (SDFA) for predicting egg production heterosis at sire level using genome-wide SNP data. Isa et al. [14] found that the genes controlling energy homeostasis and oxidative stress drove the egg production heterosis in the mid-laying periods by transcriptomic analysis of ovarian tissue. However, these findings were limited due to the single-time point sampling since numerous studies including GWAS, transcriptomic analysis and metabolomic analysis indicated that egg production was a complicated quantitative trait with dynamic genetic determinants throughout the laying periods [15–17]. Using time-series transcriptome and integrative analysis to investigate the genetic architecture of phenotypes was demonstrated to be a powerful tool [18], as well as for illustrating mechanism of plant heterosis [6].

Thus, to uncover the mechanism for egg production heterosis, we analyzed genome-wide gene expression data of hens’ ovary tissues in 5 typical laying stages for two purebreds and their reciprocal crosses. We pinpointed potential regulators and co-regulated networks and constructed gene interaction networks and their patterns to better understand the regulatory networks underlying the heterosis for egg production in chicken crossbreds.

## Materials and methods

### Experimental animals

White Leghorn chicken that introduced from Canada and Chinese indigenous breed Beijing You chicken kept in Beijing were selected to produce 4 F1 chicken groups that were 2 parental lines (WW and YY) and 2 cross lines (WY and YW). The full mating procedure has been reported previously [10]. Briefly, thirty Beijing You roosters were randomly mated with 150 Beijing You hens and 150 White Leghorn hens to generate YY and YW, respectively. Thirty White Leghorn roosters were randomly mated with another 150 White Leghorn hens and another 150 Beijing You hens to generate WW and WY, respectively. All F1 birds were hatched on the same day and housed in the identical house under the standard management conditions at the experimental farm of Institute of Animal Science, Chinese Academy of Agricultural Sciences.

### Phenotype measurements and heterosis calculation

At 18 weeks of age, birds were transferred to the hen house and raised in the individual cages providing ad libitum access to water and feed during the laying period. We measured 1,260 hens including 315 YY, 271 WW, 315 WY and 359 YW for daily egg number from age at first egg to 100 weeks of age [19]. Heterosis for egg production was calculated every 4 weeks using the formula below:

$$H= \frac{\overline{F }-(\overline{{P }_{p}}+ \overline{{P }_{m}})/2}{(\overline{{P }_{p}}+\overline{{P }_{m}})/2}$$

where $\overline{F }$, $\overline{{P }_{p}}$ and $\overline{{P }_{m}}$ represent the mean value of traits for the reciprocal crosses, WW and YY, respectively. Then, a transformed Student’s *t*-test value was calculated based on the formula below [11]:

$$t= \frac{H}{2\sqrt{\frac{\sum {({F}_{i}-\overline{F })}^{2}}{n-1}}/\left[(\overline{{P }_{p}}+\overline{{P }_{m}})\times \sqrt{n}\right]}$$

where ${F}_{i}$ is the phenotypic value of $i$^th^ bird from reciprocal crosses; $\overline{F }$ is the mean value of traits for the reciprocal crosses; $n$ is the number of crossbreds (WY or YW) used in the study. The significance of heterosis was obtained from pt() function in the R software, and the *P* value less than 0.05 was considered significant.

### Sample collection and sequencing

The ovary samples were collected at 22, 35, 46, 72 and 100 weeks of age, which represented laying stage of age at first egg (s1), peak laying stage (s2), mid-laying stage (s3), late-laying stage (s4) and prolonged-laying stage (s5), respectively. The 5 laying stages were selected according to the laying rate of the experimental population. When the laying rate of experimental population reached 5% (7.33% at 22 weeks of age), 6 chickens from WW, YY, WY and YW were selected for sampling based on the average of pubic space of each group [10]. The peak laying stage ranged from 29 to 40 weeks of age with laying rate > 80%, we sampled ovary tissues at 35 weeks of age for each breed. The persistent laying periods ranged from 41 to 79 weeks of age and were further split into the mid-laying stage sampled at 46 weeks of age and the late-laying stage sampled at 72 weeks of age, representing 70% – 80% laying rate and 60% – 70% laying rate, respectively. To prolonged the laying stage of hens to 100 weeks was put forward recently. Accordingly, we sampled tissues at 100 weeks of age. During the latter 4 stages, 6 chickens from each genetic group were selected for sampling based on the average value of egg numbers within each group. Birds were euthanized by cervical dislocation for harvesting ovary tissues, which were frozen immediately in liquid nitrogen after removing visible follicles. Total RNA of samples was isolated using the TRIzol® Reagent (Invitrogen, USA) according to the manufacturer’s instructions. A total of 120 qualified RNA samples were used for Ribo-minus strand-specific RNA sequencing to generate 150 bp paired-end sequences on the Illumina nova 6000 platform (Illumina Inc., San Diego, CA, USA).

### Quality control, mapping, and transcriptome assembly

We filtered reads that contained adaptors, more than 10% unknown nucleotides and more than 50% low-quality bases (Qphred ≤ 20). And then, high-quality clean reads with Q20 > 95% were mapped to the chicken reference genome (GRCg7b) using the Hisat2 (v2.1.0) [20]. The mapped data were assembled into transcripts using default parameters in the Stringtie (v2.1.5) [21]. The gene-level counts were counting by the featureCounts software (v2.0.3) [22]. The resulting count data was regularized log transformed using the rlog() function for the next principal component analysis (PCA), which was visualized in the Hiplot platform [23]. The expression of genes was normalized into fragments per kilobase million (FPKM). Genes with FPKM larger than 0.1 and expressed in at least 30% of all samples were considered as expressed genes, which were used for downstream analysis.

### Weighted gene co‑expression network analysis (WGCNA)

Co-expression gene modules of ovary were constructed for WW, YY, WY and YW using WGCNA in R [24], respectively. For each group, expressed genes with median absolute deviation (MAD) of expression values ranked top 50% were used in the analysis. Firstly, an appropriate “soft-thresholding” value was selected by plotting the strength of the correlation against a series of powers from 1 to 30, with a signed pairwise correlation matrix generated by Pearson’s product moment correlation. The correlation matrix was subsequently transformed into an adjacency matrix, in which nodes and edges corresponded to genes and the connection strengths between genes, respectively. Each adjacency matrix was then normalized using a topological overlap function. Genes were then clustered using average linkage hierarchical clustering methods [25]. The hierarchical cluster tree was cut into modules using the dynamic tree cut algorithm with a minimum module size of 50 genes and dendrogram cut height of 0.995. Finally, we merged modules that were highly correlated using eigengenes calculated as the first principal component of the expression profiles of each module. This was repeated until no modules were merged by clustering module eigengenes using the dissimilarity (one minus their correlation), cutting the dendrogram at the height mergeCutHeight = 0.25 and merging all modules on each branch. After obtaining modules, the correlations between a module and egg production traits were further calculated using module eigengenes (MEs) and phenotypic value, and the significance was evaluated by the corPvalueStudent() function.

In addition, the preservation of identified modules was evaluated using the modulePreservation() function implemented in WGCNA. According to the previous methods [6], we took “reference” and “test” network modules as input to calculate three statistics including density-based statistics which assessed the similarity of genes connectivity patterns between a reference network module and a test network module, separability-based statistics that examined whether test network modules remained distinct in the reference network modules, and connectivity-based statistics that were based on the similarity of connectivity patterns between genes in the reference and test networks. The Zsummary score was used to determine the preservation. Zsummary value greater than 10 indicated that the module was strongly preserved between the reference and test network modules, and a value between 2 and 10 indicated weak to moderate preservation, and a value was less than 2 indicating no preservation [26].

### Conserved co-expression network analysis

Based on the co-expression modules and available genes in each genetic group, we detected the consensus modules (CMs) underlying egg production among the WW, YY, WY and WW, YY, YW, respectively, using blockwiseConsensusModules() function in WGCNA [27]. In this scenario, a consensus adjacency matrix was created using the scaled adjacency matrix from each group dataset with consensusQuantile set to 0, and then a consensus topological overlap matrix (TOM) was generated from the consensus adjacency matrix. Consensus modules were calculated using hierarchical clustering and dynamic tree cutting with the parameters: deepSplit 2, detectCutHeight of 0.995, minModuleSize of 50 and mergeCutHeight of 0.25. Modules presenting both high correlation and similar expression profiles were merged using the mergeCloseModules() function with both cutHeight and consensusQuantile set to 0.25. The relationships between CMs and modules detected in each genetic group were calculated using fisher exact test. Gene expression patterns of the CMs across samples were represented by module eigengenes (MEs) as mentioned above. The connectivity of each gene to its corresponding module was calculated using a module membership (kME) value that was defined as the bi-weight mid-correlation between the gene expression and the corresponding ME [24]. Using each gene’s intramodular kME, we identified hub genes in the conserved network of each group [28]. The relationships between CMs in each group were analyzed by examining the bi-weight mid-correlation between MEs.

### Gene expression pattern analysis

The FPKM value was used in the analysis of gene expression pattern. The difference of gene expressions among 2 purebreds and one of crossbreds were tested by using pairwise Student’s *t*-test, and the false discovery rate (FDR) was used to determine significance of differential expression. According to the gene action modes previously reported [29], we classified gene expression into additivity (IV, X) when its expression was significantly (FDR < 0.05) different between the 2 purebreds, and the expression of the reciprocal crosses (WY or YW) was not significantly (FDR ≥ 0.05) different from the average of 2 purebreds. Gene expression of crossbreds (WY or YW) that was not significantly different from that of one purebred but significantly (FDR < 0.05) larger or smaller than that in the other purebred was regarded as dominance, which included high-parent pattern (III, XI) and low-parent pattern (V, IX). Gene expression of crossbreds that was significantly (FDR < 0.05) higher or lower than both purebreds were considered overdominance, which could be further divided into above (I) or below parent (VII) when gene expression between 2 purebreds was not significantly (FDR ≥ 0.05) different, and above high-parent (II, XII) or below low-parent (VI, VIII) when gene expression between 2 purebreds was significantly (FDR < 0.05) different.

### Function annotation analysis

Function annotation and enrichment analysis of genes were performed with Gene Ontology (GO) knowledgebase and Kyoto Encyclopedia of Genes and Genomes (KEGG) pathway database using clusterProfiler package [30] implemented in R. False discovery rate method was used to adjust the *P* values from the hypergeometric test. GO terms and KEGG pathways with adjusted *P* value < 0.05 were significantly enriched.

## Results

### Significant heterosis for egg production in chicken reciprocal crosses

**Fig. 1:**
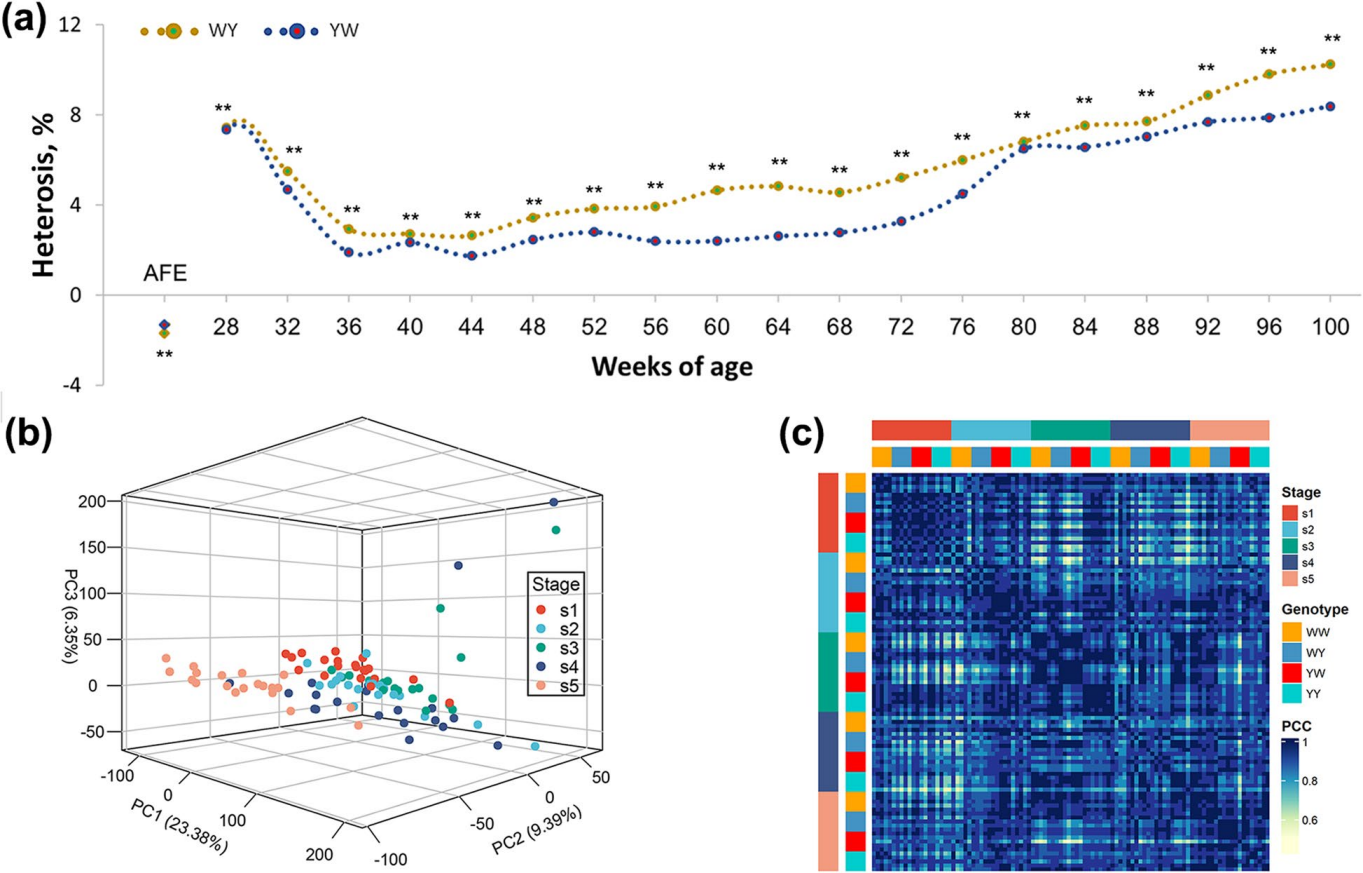
Transcriptome landscapes of ovary in chicken purebreds and crossbreds showing heterosis for egg number. **a** Continuous heterosis in the reciprocal crosses. **b** PCA for transcriptome profiles of chicken ovary in the 5 laying stages. **c** Heatmap for pairwise Pearson correlation coefficients between each pair of transcriptome profiles

We constructed the reciprocal crosses using 2 genetically distant chicken breeds (White Leghorn and Beijing You chicken) to decipher the molecular mechanism of egg production heterosis. The crossbreds (WY and YW) showed significant heterosis throughout the laying periods (Fig. 1a), which was divided into pre-peak laying stage from the age at first egg to 27 weeks (s1), peak laying stage from 28 to 40 weeks (s2), persistent laying stage from 41 to 72 weeks (further split into mid laying stage from 41 to 55 weeks (s3) and late laying stage from 56 to 79 weeks (s4)) and prolonged laying stage from 80 to 100 weeks (s5) based on the physiological status and egg production rate of hens. Compared to mid‐parent value (MPV), the crossbreds showed 2.88 days (WY) and 2.23 days (YW) earlier for the age of first egg, respectively. During s1, the average heterosis for egg production was 7.44% and 7.36% for WY and YW, respectively. During persistent laying period (s2–s4), the difference of laying rate between 2 purebreds were getting smaller, resulting in a high mid-parent value. Hence, the heterosis decreased in both crossbreds with the average heterosis 4.02% and 2.67%, respectively. During s5, the heterosis increased to 10.24% and 8.38% for WY and YW, respectively. Overall, the crossbreds laid 33.28 and 27.24 more eggs than the MPV (Table S1).

Subsequently, the 5 representative laying stages, including 22, 35, 46, 72 and 100 weeks of age were selected to harvest the ovary tissues for transcriptomic sequencing. The RNA sequencing on the 120 ovarian tissues for purebreds and crossbreds (six biological replicates for each stage and each group) generated 1.31 trillion data. As shown in the Fig. S1, these samples were evaluated for quality with average Q30 of 94.49%. By mapping to the chicken reference genome (GRCg7b), we obtained average mapping rate of 94.54%. Principal component analysis (PCA) showed the transcriptome profile of s5 was separated from the other 4 stages, whereas s1 to s4 were interacted with each other, suggesting the relatively large transcriptomic changes in the prolonged laying period (Fig. 1b). Except for the s2, the WW and YY could be separated in each stage, while the crossbreds were not clearly clustered (Fig. S2). Furthermore, the genome-wide gene expression in both purebreds and the crossbreds remained closely correlated with a value lager than 0.56 supported by the pairwise Pearson correlation coefficients (Fig. 1c), indicating highly conserved transcriptional features in the purebreds and crossbreds during the whole laying periods.

### Gene regulatory networks correlate with the egg production

**Fig. 2:**
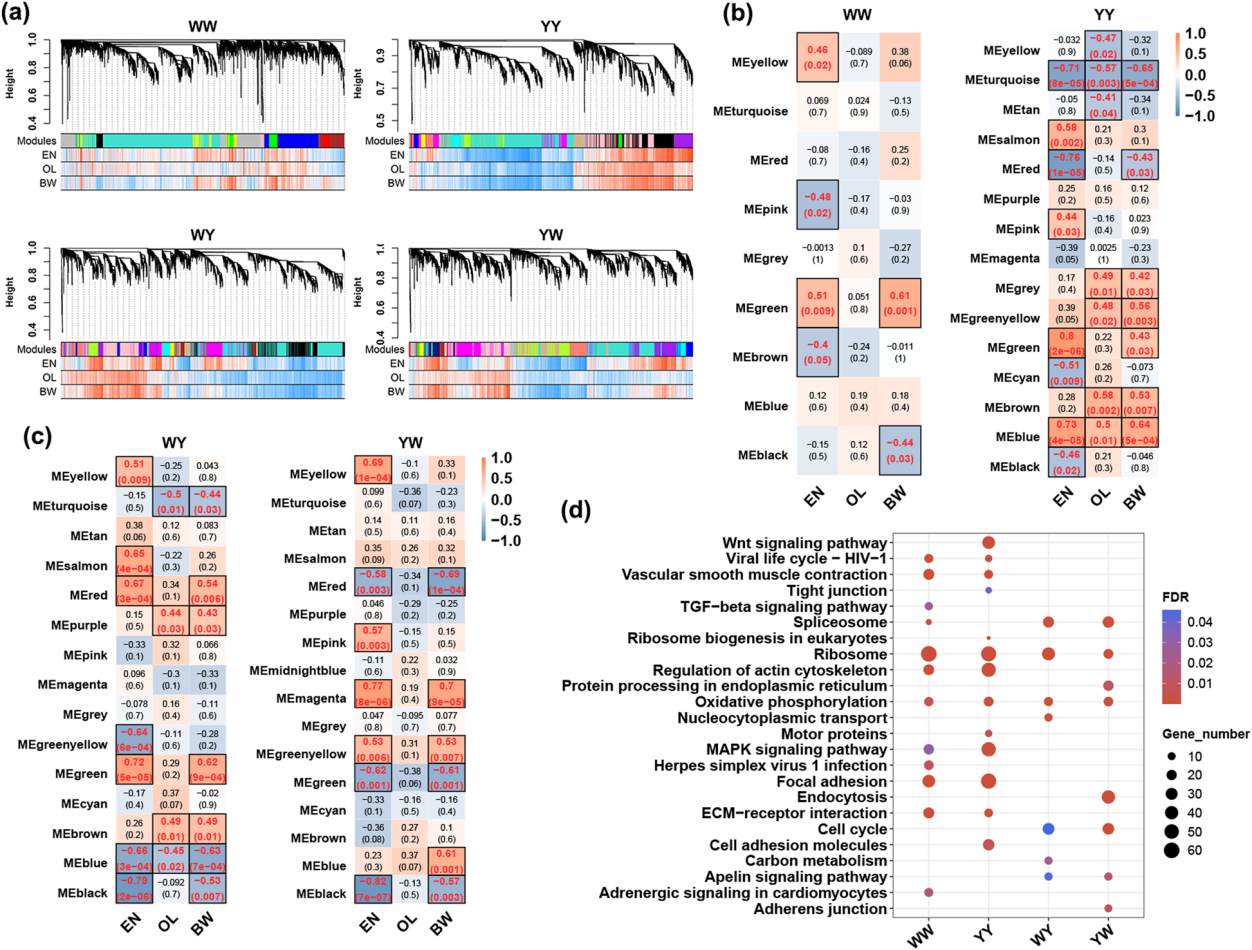
The weighted gene co-expression network analysis (WGCNA) for chicken reciprocal crosses (WY and YW) and their parents (WW and YY). **a** Gene dendrogram obtained by average linkage hierarchical clustering for each genetic group. The color row underneath the dendrogram shows the module assignment and expression heatmap of genes corresponding to the assignment. **b** and **c** The module-trait relationships for parents and reciprocal crosses, respectively. Each row represents module eigengenes, and each column represents a trait including egg number (EN), oviduct length (OL) and body weight (BW). The correlation coefficients and *P* value (in the parentheses) were presented in the cell. **d** Significant KEGG pathways enriched by genes harbored in the modules that significantly correlated with egg number

Using the high-quality transcriptomic data sequenced in the 5 laying stages, we conducted the weighted gene co-expression network analysis (WGCNA) and correlated the phenotypes with the co-expressed gene modules for both purebreds and crossbreds to identified the gene regulatory networks related to the egg production. We selected genes with MAD ranked top 50% to construct a signed, scale-free network for each genetic group. The appropriate soft-thresholding power (β) for network construction were estimated as 24, 16, 16 and 16 for WW, WY, YW and YY, respectively (Fig. S3). We obtained 11,646 genes harbored in 9 modules, 11,654 genes harbored in 15 modules, 11,717 genes harbored in 16 modules and 11,718 genes harbored in 15 modules for WW, YY, WY and YW, respectively (Fig. 2a). The correlations between genes and investigated traits, including egg number (EN), oviduct length (OL) and body weight (BW) was diverse among 4 genetic groups. For example, the correlations in WW were largely different from that in YY (Fig. 2a), suggesting the breed difference in terms of global gene expressions and their interactions with traits. We further calculated the correlations between gene modules and traits, revealing 4 (2 positively modules and 2 negatively modules) and 8 modules (4 positively modules and 4 negatively modules) were significantly correlated with EN (EN-related) for WW and YY, respectively. Among these modules, the EN-related green module (MEgreen) of WW was also correlated with BW, while the EN-related MEyellow, MEpink and MEbrown were uniquely related to EN. The EN-related red and green modules of YY were also correlated with BW, and the EN-related turquoise and blue modules of YY were related to all 3 traits (Fig. 2b). In the 2 crossbreds, we detected 7 modules each associated with EN (Fig. 2c). Regarding the WY, 2 of 4 positively EN-related modules (MEred and MEgreen) were also related to BW, and the blue EN-module was correlated with the three traits. For the YW, 2 of 4 positively EN-related modules (MEmagenta and MEgreenyellow) were also correlated with BW, and all three negatively EN-related modules (MEred, MEgreen and MEblack) were also correlated with BW.

We then tested for gene enrichment of EN-related modules against GO and KEGG pathways databases to detect the biological process (BP) and pathways involved for the 4 genetic groups. In total, we found that genes harbored in the EN-related modules of WW, YY, WY and YW were significantly enriched in 38, 64, 33 and 15 BPs, respectively (Table S2a). After overlapping, a total of 21 BPs, such as cell adhesion, oxidative phosphorylation, peptide metabolic process and cell migration were shared by WW and YY, while other BPs accounting for 67% of total BPs were breed-specific (Fig. S4). In terms of KEGG, we identified 15, 25, 22 and 16 KEGG pathways were significantly enriched by EN-related module genes for WW, YY, WY and YW, respectively (Table S2b). By filtering pathways with gene ratio less than 5%, we found that the ribosome and oxidative phosphorylation were shared by 4 genetic group. Multiple pathways, such as focal adhesion, ECM-receptor interaction, vascular smooth muscle contraction and regulation of actin cytoskeleton were shared by 2 purebreds. The TGF-beta signaling pathway and Wnt signaling pathway were specifically identified for WY and YW, respectively. Notably, the apelin signaling pathway, cell cycle and spliceosome were enriched by module genes in the 2 crossbreds (Fig. 2d). These results suggested that divergent biological processes and pathways were involved in the egg production among purebreds and crossbreds, and the common networks among different genetic groups were potential regulators for the formation of egg production heterosis.

### Highly preserved gene regulatory networks among the crossbreds and purebreds

Given the close correlations of gene expressions and common KEGG pathways/GO terms enriched by WGCNA module genes, we assessed the conservation of gene co-expression networks between the crossbreds and the purebreds by conducting 100 times of permutation tests. By projecting the WW transcriptome dataset onto the network of YY, WY and YW, all modules were strongly preserved with Zsummary > 10 (Fig. S5a). When we took modules identified in YY as a reference network, we found that all modules in the crossbreds were at least weakly (Zsummary > 2) preserved (Fig. S5b). Moreover, highly preserved gene regulatory networks were found between the 2 crossbreds by setting WY as a reference network (Fig. S5c). The conserved gene networks were corresponded to the highly correlated transcriptional architecture underlying the transcriptome profile of ovary in the purebreds and crossbreds (Fig. 1c). Subsequently, we investigated transcriptional differences in the core gene regulatory networks, which were promisingly related to the significant heterosis for egg production in the crossbreds.

### Consensus gene regulatory networks and their expression patterns between the crossbreds and purebreds

**Fig. 3:**
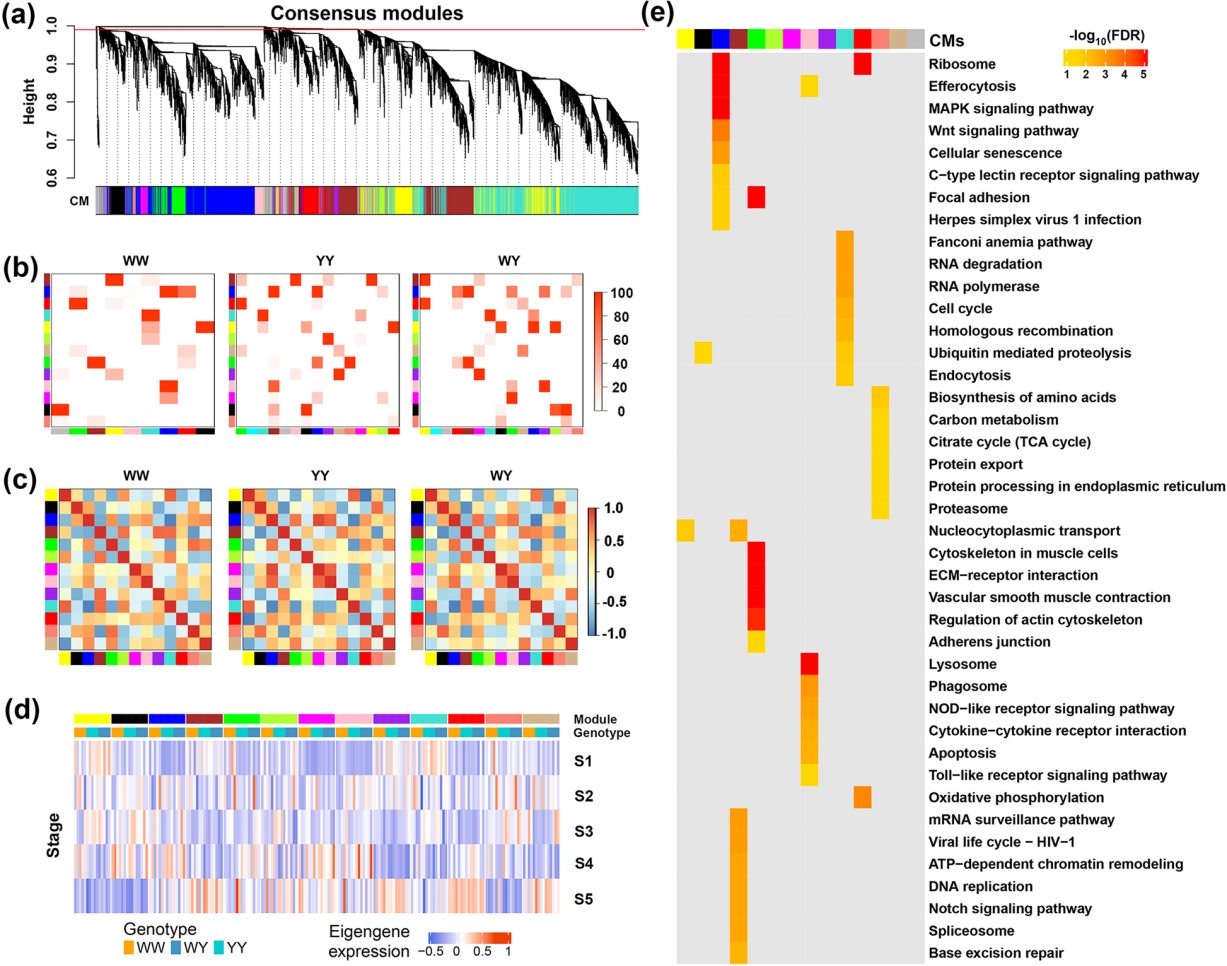
The consensus and divergence of gene co-expression networks between the crossbred WY and its parents (WW and YY) during the 5 laying stages. **a** Hierarchical cluster dendrogram showing consensus gene co-expression modules (CMs) among WW, YY and WY. The red line above is the cut height (0.995) for CM identification. CM colors are labeled independent of the separately identified modules in each group. **b** The relationships between global and group specific co-expression networks. The vertical axis represents CMs. The horizontal axis represents modules within each group. The correlation is tested by Fisher exact test. The legend is showing −log10 transformed *P* value. **c** The relationships among eigengenes of CMs in each group. **d** The expression patterns of eigengenes of CMs in WW, YY and WY during 5 laying stages. Blue and red represent lesser and greater expression, respectively. **e** Heatmap showing KEGG pathways significantly enriched by genes in each CM. Color intensity represents the significance of enrichment

The heterosis difference (Fig. 1a) and different EN-related WGCNA modules (Fig. 2) between 2 crossbreds suggested that divergent regulatory networks contributed to the egg production heterosis. Therefore, we analysis the patterns of conserved gene regulatory networks for WY and YW, respectively. Based on the gene modules previously detected, we firstly constructed a conserved gene co-expression network underlying 5 laying stages among WW, YY and WY. As showing in the Fig. 3a, a total of 10,666 genes were assigned to the conserved network. They were clustered in 14 CMs, consisting of 100–3,100 co-expressed genes (Fig. 3a and Table S3a), which were expressed consistently in different genetic groups. We then created CM eigengenes network and clustered CM eigengenes for each genetic group. The dendrogram showed that the CM clusters of WY were different from that of purebreds (Fig. S6a). The relationships between CMs and WGCNA modules previously detected showed that the modules in each genetic group (genotype-specific modules) were split into multiple CMs. For example, the EN-related green module was associated with blue, red and tan CMs in WW, and it was associated with brown, red and tan CMs in YY. The EN-related black module was associated with yellow, black and pink CMs in WY (Fig. 3b). We further analyzed inter-module correlations among the 13 CMs (excluding grey modules) in each genetic group by generating an eigengene network. The pair-wised correlation coefficients among CM eigengenes indicated that networks of WY were partially similar to that of 2 purebreds in some modules (Fig. 3c). Furthermore, we compared the expression pattern of eigengenes in the CMs, and observed differing expression patterns of eigengenes between crossbred and purebreds across 5 laying periods (Fig. 3d). For example, the expression levels of blue CM eigengenes increased over s1–s4 and then decreased in s5 across three genetic groups, while the expression levels of turquoise CM eigengene decreased over s1–s4 and then increased in s5 across three genetic groups. KEGG pathway analysis indicated that genes of the blue CM were significantly enriched in ribosome, efferocytosis, MAPK signaling pathway, Wnt signaling pathway, cellular senescence, focal adhesion, etc. (Fig. 3e). The genes of turquoise CM were enriched in the RNA degradation, cell cycle and ubiquitin mediated proteolysis and endocytosis were decreased during laying periods. The brown CM genes that enriched in the mRNA surveillance pathway, Notch signaling pathway and spliceosome were decreased in s1–s3 and then increased in s4–s5. The pink CM genes that enriched in the immune-related pathways, such as lysosome, phagosome, NOD-like receptor signaling pathway, cytokine-cytokine receptor interaction, apoptosis and toll-like receptor signaling pathway, were highly expressed in s1, s3 and s5 while lowly expressed in s2 and s4. Moreover, the genes of red CM that overrepresented in the ribosome and oxidative phosphorylation were highly expressed in s5 (Fig. 3e and Table S4a). Notably, the eigengene expression of CMs in the crossbreds was more specific compared to the purebreds, suggesting a genotype-specific perturbation of gene expression levels in these CMs (Fig. 3d).

Using same pipeline, we analyzed the transcriptional differences that were associated with the egg production heterosis among WW, YY and YW. We identified 10,640 genes were cluster into 14 CMs (Table S3b and Fig. S7a). Genotype-specific modules were also correlated with multiple CMs, for example, green module in YW was correlated with yellow, purple and pink CMs (Fig. S7b). The correlation heatmap among CMs showed that the expression networks of YW were specifically similar to that of 2 purebreds in some modules (Fig. S7c). We found CMs like blue decreased over s1–s4 and then increased in s5 in three genetic groups (Fig. S7d). Genes harbored in the blue CM were significantly enriched in the cell cycle, DNA replication, progesterone-mediated oocyte maturation and so on (Fig. S7e and Table S4b). Based on gene expression results from the 2 crossbreds, we deduced that the dynamic gene expressions and interactions in various pathways were associated with egg production heterosis in the crossbreds.

### Dominant gene expression for conserved biological pathways in the crossbreds

**Fig. 4:**
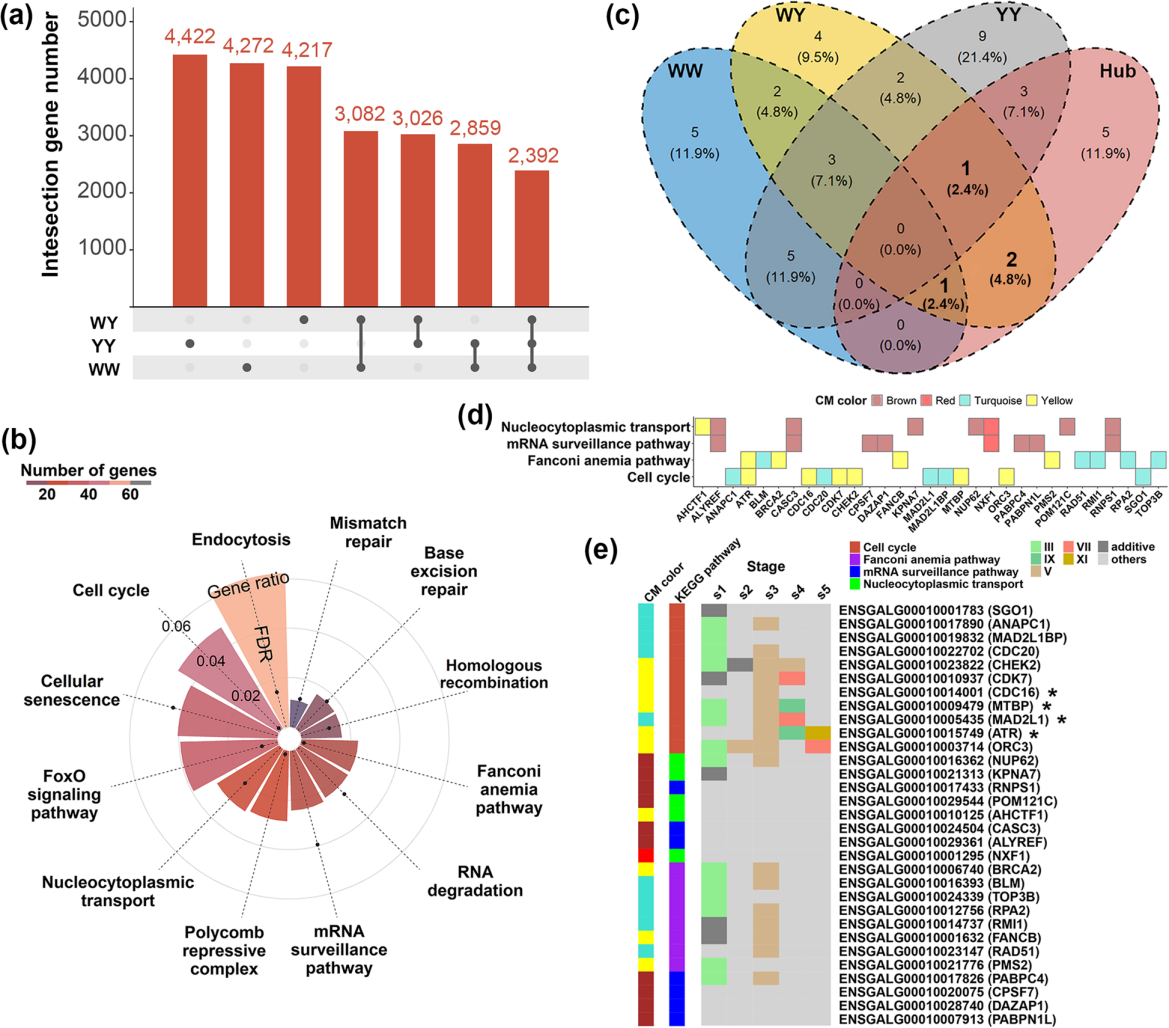
Hub genes in the preserved gene co-expression network underlying the dynamic egg production heterosis. **a** Upset plot showing the common hub genes among WW, YY and WY. **b** KEGG pathways enrichment analysis for the common hub genes. The color bar represents the ratio of gene numbers in each pathway, and the black dot represents the significance (adjusted P value) of the KEGG pathway. **c** Venn diagram showing the overlapped KEGG pathways that enriched by EN-related module genes in WW, EN-related module genes in WY, EN-related module genes in YY and hub genes in CMs. **d** The involved genes in the common pathways shared by EN-related module genes in WY and hub genes in CMs. **e** The complementary differential expression patterns during 5 laying stages for hub genes involved in the cell cycle. The cell colors represent differential expression patterns, and the colors on the left represent the corresponding CM and KEGG pathway to which each gene belongs, respectively. Genes with asterisk indicate that they are detected in both crossbreds

**Fig. 5:**
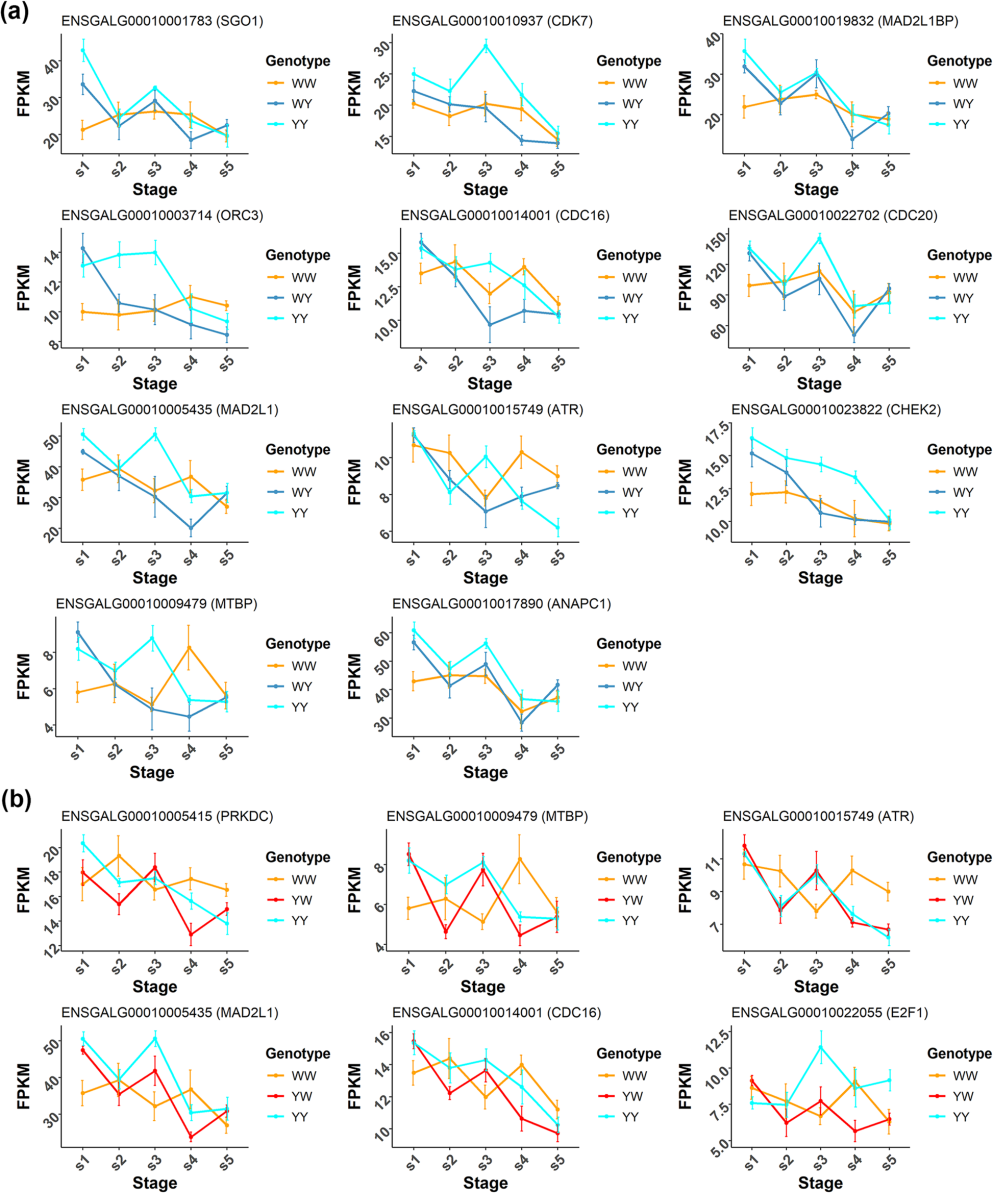
Expression levels of representative hub genes involved in the cell cycle process for WY (**a**) and YW (**b**) during 5 laying stages, respectively. Data are mean FPKM ± SE in each group at each stage

To identify key regulators that may participate in the dynamic regulation of heterosis in the crossbreds, we screened hub genes that were highly connected to other genes in the conserved gene co-expression networks for WY and YW, respectively. We calculated the module membership value (kME) for each gene, from which we identified 4,272, 4,422 and 4,217 hub genes (kME > 0.70, *P* < 1e-5) in WW, YY and WY, respectively (Fig. 4a and Table S5). Among these hub genes, a total of 2,392 genes were shared by three genetic groups, indicating that these genes have preserved functional features across three groups. Function enrichment analysis revealed that these shared hub genes were significantly (FDR < 0.05) overrepresented in GO biological processes related to cell cycle, DNA damage response, macromolecule catabolism and RNA catabolism (Table S6a). KEGG pathway analysis showed that the shared hub genes were enriched in the endocytosis, cell cycle, cellular senescence, FoxO signaling pathway, etc. (Fig. 4b and Table S7a), some of which were also enriched by genes in the EN-related modules. As showing in Fig. 4c, 1, 4 and 4 pathways were overlapped with enrichment pathways of hub genes for WW, YY and WY, respectively. The 4 overlapped pathways for the WY were cell cycle, nucleocytoplasmic transport, mRNA surveillance pathway and Fanconi anemia pathway, among which common genes were found between nucleocytoplasmic transport and mRNA surveillance pathway and between cell cycle and Fanconi anemia pathway, respectively (Fig. 4d). By focusing on the 31 unique genes, we further profiled their expression patterns. The dominant expression patterns were found for genes overrepresented in the cell cycle and Fanconi anemia pathway (Fig. 4e). The cell cycle pathway regulates cell division and proliferation including cell growth and DNA replication, and the Fanconi anemia pathway is made up of 22 proteins that work together to repair damaged DNA and assist with DNA replication and other cellular processes. The genes involved in the cell cycle were mostly expressed in high-parent patterns during s1, and then they were transformed into low-parent patterns during s3. The expression levels of genes were higher in YY than that in WW (Fig. 5a), and the number of dominance genes in s2, s4 and s5 were less than that in s1 and s3. Among these genes, *ORC3* and *CHEK2* were also dominantly expressed in low-parent pattern during s2 and s4, respectively. *CDK7* and *MAD2L1* were expressed in below-parent pattern, while *MTBP* and *ATR* were in low-parent pattern. During s5, *OCR3* and *ATR* were expressed in below-parent pattern and high-parent pattern, respectively. *CHEK2* encodes a protein that functions as a regulator of the cell cycle as well as a tumor suppressor. The protein is activated in the presence of DNA damage to prevent the entry into mitosis. *MAD2L1* accumulates early in oocyte maturation and affects cell proliferation and cell cycle progression.

In the combination of WW, YY and YW, we identified 1,866 common hub genes, which were enriched in the GO BPs related to cell cycle and macromolecule catabolic process (Table S6b) and KEGG pathways including efferocytosis, cellular senescence, cell cycle and FoxO signaling pathway (Fig. S8 and Table S7b). Among these pathways, cell cycle was the only KEGG pathway overlapped with pathways enriched by EN-related module genes (Fig. 4c). Again, we profiled expression patterns for 6 common pathway genes and found that the 4 identical genes including *MAD2L1*, *CDC16*, *MTBP* and *ATR* were additively expressed or dominantly expressed in high-parent model during s3 (Fig. S8c). This is probably the underlying factors leading to the heterosis difference between 2 crossbreds. Another 2 genes including *PRKDC* and *E2F1* were expressed in low-parent pattern during s4 and during s3 and s5, respectively. Similar low-parent expression pattern between WY and YW for *MTBP* and *ATR* were found during s4, while the expressions were higher in WW than that in YY (Fig. 5b). *E2F1* was one of transcription factors of E2F family, playing an important role in the control of the cell cycle, cell proliferation and differentiation. *PRKDC* encodes the DNA-dependent protein kinase catalytic subunit (DNA-PKcs) protein, which plays an important role in nonhomologous end joining of DNA double-strand breaks and is also closely related to the establishment of central immune tolerance and the maintenance of chromosome stability. These results supported the key roles of hub genes involved in the cell cycle pathway played in egg production heterosis during 5 laying stages in the crossbred chickens.

## Discussion

Unlike to the crop breeding, crossbreds are produced by crossing pure lines that are usually not homozygous in animals. The mechanisms of heterosis were complicated and need a more systematic dissection. Nevertheless, different animal breeds possessed distinct genetic characteristics, which could be stably inherited by the next generation within breed. In the current study, the 2 parental lines used in the study were genetically distant [31]. The White Leghorn chicken was continuous under close selection for egg production in Canada and after imported to Beijing. The Beijing You chicken was famous for its appearance with crest, beard and feather leg. The 2 breeds possessed significant difference in terms of egg production and quality, growth, behaviors etc. These made the research more enlightening. Previous studies demonstrated that regulatory genes and networks expressed in dominance, overdominance or epistasis patterns explained the heterosis for growth and reproduction traits in animals [8, 32]. However, there remain many limitations and lacks, especially for economic traits in chickens. For example, most studies were conducted with single-stage tissue sampling, resulting in transcriptional noise and false positive results. In addition, previous transcriptome analyses focus on differentially expressed genes (DEGs) with big changes, but it is largely unknown whether these genes are coordinator genes or not. How these DEGs collaborate with each other is difficult to be revealed. Here, we started from identifying the key genetic factors related to the egg production within each genetic group, and then profiled temporally dynamic genome-wide gene expression during laying periods in the crossbreds and their parents. Based on the results of 2 parts, we identified gene co-expression network differences and hub genes between the crossbreds and their parents that underlay the dynamic regulation of egg production heterosis.

Our analyses revealed highly preserved gene regulatory networks shared by the crossbreds and purebreds. The functions of hub genes in the regulatory networks were consistent with the reported signaling pathways that related to the follicle development and selection in the ovary, such as cell cycle [33], cellular senescence [34], endocytosis [35] and Foxo signaling pathway [36]. For the crossbreds, we found that common hub genes in the cell cycle pathway were associated with egg production heterosis. This was supported by previous studies, which reported that the cell cycle pathways were highly enriched in oocytes from primary follicles and MII oocytes to regulate the proliferation of granulosa cells and ovarian follicular growth and ovulation [37, 38], suggesting critical roles of the biological processes in egg production. Notably, the dynamic expression of common genes between 2 crossbreds was in a contrary pattern during the mid-laying periods (s3). Obviously, the divergent expression pattern could be attributed to alteration in the regulation of allele-specific gene expression that was highly dependent on the environmental context [39]. The multi-omics studies need to be further conducted to elucidate the intriguing phenomenon [40]. Regarding the function of common genes, reduced expression levels of *MAD2L1* have been shown to cause a shortened duration of meiotic I and meiotic spindle abnormalities, promoting oocyte maturation [41, 42]. This was consistent with that *MAD2L1* expression was downregulated during s3 to s4 for the 2 crossbreds, indicating that *MAD2L1* plays an important role in chicken egg production heterosis during persistent laying periods.

The dominant expressions of genes have been proposed as a well-known mechanism for heterosis, but the dynamic dominance of genes associated with heterotic phenotypes was rarely studied. In the *Arabidopsis*, the complementary dominant gene expression patterns between regulatory genes involved in the photosynthesis and cell division pathways led to the biomass heterosis in different stages of development [6]. Here, we didn’t identify potential signaling pathways complementary to the cell cycle pathway due to the single tissue sampling. However, the complementary expression of cell cycle pathway genes in the 5 laying stages was found in the reciprocal crosses. Before laying the first egg (s1), hub genes in the cell cycle pathway of crossbred chickens exhibited YY-like higher expression, whereas they exhibited WW-like lower expression in the persistent laying stages (s3 and s4). During s1, chicken gonads developed rapidly to preserved sufficient primary follicles for the egg laying. The precocity (laying first egg at early age) was reported to be a negative factor affected the chicken egg production and its rate [43]. Hence, the crossbred chickens inherited more from parent YY to save energies and follicles for a higher egg production. During the persistent laying periods, the crossbred chickens switched the gene expression direction to WW, which preserved the ovarian function for persistently high egg yielding. This implied that the dynamic gene expression during laying periods contributed to the heterosis. Moreover, the functions of dynamically expressed genes including *MAD2L1* in both crossbreds, *CHEK2* in WY and *E2F1* in YW supported the implication. For example, the specific inhibition of *CHEK2* contributing to maintain ovary function has been demonstrated in human beings [44]. The knockdown of *E2F1* promoted cell growth, follicular development and estradiol synthesis by increasing *CYP19A1* promoter activity in the porcine granulosa cell [45]. It is important to acknowledge certain limitations of the present study, such as the precise mechanism of candidate genes affected the egg production heterosis by regulating the ovarian function, which need further experiments within and between chicken breeds.

## Conclusion

In summary, the continuous heterosis for egg production was analyzed during the whole laying periods. We profiled the dynamic gene expression of ovary and identified preserved co-expression networks relevant to the egg production in the crossbreds and their parents. The complementary dominance of regulatory genes involved in the cell cycle were firstly found to contribute to the egg production heterosis. Our findings laid valuable foundation for practical use of the dominance model in crossbreeding. The gene co-expression networks and candidate genes could accelerate future researches on the regulatory mechanisms for egg production and its heterosis.

## Supplementary Information

Additional file 1: Fig. S1 The summary of sample sequence statistics including clean reads, clean bases, Q20, Q30, GC content and overall mapping rate. Fig. S2 The PCA for transcriptome profiles of purebreds and crossbreds in each laying stage. Fig. S3 Scale independence and mean connectivity for purebreds and crossbreds using various soft threshold powers. Fig. S4 Venn diagram showing the overlapped GO terms enriched by EN-related module genes in each genetic group. Fig. S5 Pairwise module preservation of gene co-expression networks between the crossbreds and their parents. Dashed red and blue lines represent the Zsummary thresholds for strong (Zsummary > 10) and weak to moderate (2 < Zsummary < 10) preservation levels, respectively. Colored dots represent the corresponding modules in the reference network, and the module size is the number of overlapped genes within each reference module. Fig. S6 The cluster profiles of consensus modules (CMs) and group-specific modules for crossbred WY (a) and crossbred YW (b) compared to their parents, respectively. Fig. S7 The consensus and divergence of gene co-expression networks between the crossbred YW and its parents (WW and YY) during the 5 laying stages. (a) Hierarchical cluster dendrogram showing consensus gene co-expression modules (CMs) among WW, YY and YW. The red line above is the cut height (0.995) for CM identification. CM colors are labeled independent of the separately identified modules in each group. (b) The relationships between global and group specific co-expression networks. The vertical axis represents CMs. The horizontal axis represents modules within each group (c) The relationships among eigengenes of CMs in each group. (d) The expression patterns of eigengenes of CMs in WW, YY and YW during 5 laying stages. Blue and red represent lesser and greater expression, respectively. (e) Heatmap showing KEGG pathways significantly enriched by genes in each CM. Color intensity represents the significance of enrichment. Fig. S8. Hub genes in the preserved gene co-expression network underlying the dynamic egg production heterosis. (a) Upset plot showing the common hub genes among WW, YY and YW. (b) KEGG pathways enrichment analysis for the common hub genes. The color bar represents the ratio of gene numbers in each pathway, and the black dot represents the significance (adjusted *P* value) of the KEGG pathway. (c) The differential expression patterns during 5 laying stages for hub genes involved in the cell cycle. The cell colors represent differential expression patterns, and the colors on the left represent the corresponding CM and KEGG pathway to which each gene belongs, respectively.

Additional file 2: Table S1 The summary of population phenotypic value in the 5 sampling weeks. Table S2a Significant GO terms enriched by genes in the modules related to the egg number for purebreds and crossbreds; Table S2b Significant KEGG pathways enriched by genes in the modules related to the egg number for purebreds and crossbreds. Table S3a The consensus genes and their connectivity (kME) among WW, WY and YY; Table S3b The consensus genes and their connectivity (kME) among WW, YW and YY. Table S4a Significant KEGG pathways enriched by genes in the consensus modules (CMs) detected for WW, YY and WY; Table S4b Significant KEGG pathways enriched by genes in the consensus modules (CMs) detected for WW, YY and YW. Table S5a Hub genes of WW screened by module membership value (kME) and its *P* value; Table S5b Hub genes of YY screened by module membership value (kME) and its *P* value; Table S5c Hub genes of WY screened by module membership value (kME) and its *P* value. Table S6a Significant GO terms enriched by hub genes shared by WW, YY and WY; Table S6b Significant GO terms enriched by hub genes shared by WW, YY and YW. Table S7a Significant KEGG pathways enriched by hub genes shared by WW, YY and WY; Table S7b Significant KEGG pathways enriched by hub genes shared by WW, YY and YW.

## Data Availability

The RNA-seq data sets have been deposited in the National Genomics Data Center database (https://ngdc.cncb.ac.cn/, NGDC) with the BioProject number PRJCA021760 and PRJCA029048.

## Abbreviations

BP: Biological process
BW: Body weight
CMs: Consensus modules
DEGs: Differentially expressed genes
EN: Egg number
FDR: False discovery rate
FPKM: Fragments per kilobase million
GO: Gene Ontology
KEGG: Kyoto Encyclopedia of Genes and Genomes
kME: Module membership
MAD: Median absolute deviation
MEs: Module eigengenes
MPV: Mid‐parent value
OL: Oviduct length
PCA: Principal component analysis
TOM: Topological overlap matrix
WGCNA: Weighted gene co-expression network analysis
WW: Purebred White Leghorn chicken
WY: Crossbred generated by crossing White Leghorn rooster and Beijing You hen
YW: Crossbred generated by crossing Beijing You rooster and White Leghorn hen
YY: Purebred Beijing You chicken
